# Two weeks of moderate-intensity continuous training, but not high-intensity interval training, increases insulin-stimulated intestinal glucose uptake

**DOI:** 10.1152/japplphysiol.00431.2016

**Published:** 2017-02-09

**Authors:** Kumail K. Motiani, Anna M. Savolainen, Jari-Joonas Eskelinen, Jussi Toivanen, Tamiko Ishizu, Minna Yli-Karjanmaa, Kirsi A. Virtanen, Riitta Parkkola, Jukka Kapanen, Tove J. Grönroos, Merja Haaparanta-Solin, Olof Solin, Nina Savisto, Markku Ahotupa, Eliisa Löyttyniemi, Juhani Knuuti, Pirjo Nuutila, Kari K. Kalliokoski, Jarna C. Hannukainen

**Affiliations:** ^1^Turku PET Centre, University of Turku, Turku, Finland;; ^2^Medicity Research Laboratory, University of Turku, Turku, Finland;; ^3^Department of Cell Biology and Anatomy, Institute of Biomedicine, University of Turku, Turku, Finland;; ^4^Turku PET Centre, Turku University Hospital, Turku, Finland;; ^5^Department of Radiology, Turku University Hospital, Turku, Finland;; ^6^Paavo Nurmi Centre, Turku, Finland;; ^7^Turku PET Centre, Abo Akademi University, Turku, Finland;; ^8^Research Centre of Applied and Preventive Cardiovascular Medicine, University of Turku, Turku, Finland;; ^9^Department of Biostatistics, University of Turku, Finland; and; ^10^Department of Endocrinology, Turku University Hospital, Turku, Finland

**Keywords:** intestine, intestinal metabolism, high-intensity interval training, moderate-intensity continuous training, exercise, positron emission tomography

## Abstract

Similar to muscles, the intestine is also insulin resistant in obese subjects and subjects with impaired glucose tolerance. Exercise training improves muscle insulin sensitivity, but its effects on intestinal metabolism are not known. We studied the effects of high-intensity interval training (HIIT) and moderate-intensity continuous training (MICT) on intestinal glucose and free fatty acid uptake from circulation in humans. Twenty-eight healthy, middle-aged, sedentary men were randomized for 2 wk of HIIT or MICT. Intestinal insulin-stimulated glucose uptake and fasting free fatty acid uptake from circulation were measured using positron emission tomography and [^18^F]FDG and [^18^F]FTHA. In addition, effects of HIIT and MICT on intestinal GLUT2 and CD36 protein expression were studied in rats. Training improved aerobic capacity (*P* = 0.001) and whole body insulin sensitivity (*P* = 0.04), but not differently between HIIT and MICT. Insulin-stimulated glucose uptake increased only after the MICT in the colon (HIIT = 0%; MICT = 37%) (*P* = 0.02 for time × training) and tended to increase in the jejunum (HIIT = −4%; MICT = 13%) (*P* = 0.08 for time × training). Fasting free fatty acid uptake decreased in the duodenum in both groups (HIIT = −6%; MICT = −48%) (*P* = 0.001 time) and tended to decrease in the colon in the MICT group (HIIT = 0%; MICT = −38%) (*P* = 0.08 for time × training). In rats, both training groups had higher GLUT2 and CD36 expression compared with control animals. This study shows that already 2 wk of MICT enhances insulin-stimulated glucose uptake, while both training modes reduce fasting free fatty acid uptake in the intestine in healthy, middle-aged men, providing an additional mechanism by which exercise training can improve whole body metabolism.

**NEW & NOTEWORTHY** This is the first study where the effects of exercise training on the intestinal substrate uptake have been investigated using the most advanced techniques available. We also show the importance of exercise intensity in inducing these changes.

## Abstract (precis)

This is the first study where the effects of exercise training on the intestinal substrate uptake have been investigated using the most advanced techniques available. We also show the importance of exercise intensity in inducing these changes.

the intestine is a large organ and a major determinant of whole body energy homeostasis through its control over nutrient absorption and release of gut hormones during digestion (6). Evidence demonstrating the potential role of the intestine in the pathogenesis of obesity and insulin resistance is rapidly increasing. In type 2 diabetes, there is a continuous deterioration of intestinal endocrine function (16), and alterations in the intestinal microbiota content have been shown to be associated with the development of insulin resistance in humans and animals (8, 9, 26). Splanchnic glucose uptake (GU) accounts for up to 60% of total glucose metabolism after an oral glucose load. In insulin resistance, splanchnic GU is impaired and plays a role in the pathogenesis of hyperglycemia in type 2 diabetes (10, 27). Our laboratory has previously shown that tissue-specific intestinal GU from circulation into enterocytes is impaired in the insulin-stimulated state, i.e., intestinal insulin resistance exists, in obese and type 2 diabetic subjects (29). The role of intestinal insulin resistance in the pathology of type 2 diabetes is unclear; however, it has been suggested that intestinal insulin resistance leads to abnormalities in the signaling mechanism responsible for the GLUT2-mediated GU in the small intestine, particularly in the jejunum, leading to increased transepithelial or lumen to blood glucose exchange, causing hyperglycemia (3).

Regular exercise training enhances skeletal muscle insulin sensitivity (11, 20, 23, 35) in working muscles. Exercise training also enhances the regulation and utilization of lipids in the skeletal muscle (13, 19, 22, 42). The training-induced adaptations in muscle substrate metabolism and oxidative capacity lead to improvements in the whole body metabolism and insulin sensitivity. Although muscle is widely studied, previous data about the effects of exercise on abdominal organs concern mainly the liver and pancreas, and data are limited about the effects of exercise on intestine (28, 33, 36). Thus it is not known whether exercise training could enhance intestinal substrate metabolism, and whether any possible changes would be reflected in the insulin sensitivity of the whole body.

Our laboratory has previously shown that 2 wk of low-volume, high-intensity interval training (HIIT) and moderate-intensity continuous training (MICT) increase both aerobic capacity and whole body and main working skeletal muscle insulin-stimulated GU in sedentary, middle-aged men (7). In the present study, using the intestine data from this same clinical trial (NCT01344928), our aim was to quantify the effects of exercise on tissue-specific, insulin-stimulated glucose and fasting free fatty acid uptake (FFAU) from circulation into the intestine (duodenum, jejunum, and colon) using positron emission tomography (PET) and radiotracers 2-[^18^F]fluoro-2-deoxy-d-glucose (FDG) and 14(*R*,*S*)-[^18^F]fluoro-6-thia-heptadecanoic acid (FTHA) before and after HIIT and MICT. We hypothesized that the higher training volume instead of the intensity would strain the intestinal metabolism more and thus lead to the increased intestinal insulin-stimulated GU and decreased FFAU after MICT compared with HIIT. Additionally, to explore possible mechanisms behind the changes in intestinal GU and FFAU, we also studied healthy Wistar rats, which underwent corresponding HIIT and MICT interventions, and analyzed the intestinal protein expression of GLUT2 and CD36. We hypothesized that training would increase the expression of GLUT2 and CD36 in enterocytes more after MICT than HIIT.

## MATERIALS AND METHODS

### 

#### Subjects.

Twenty-eight middle-aged, sedentary individuals were recruited and randomized into two groups; one with 2 wk of HIIT and the other with 2 wk of MICT. The subjects were nonobese [aged 40–55 yr, peak O_2_ uptake (V̇o_2peak_) < 40 ml·kg^−1^·min^−1^] and had no previous experience of active exercise training. The inclusion and exclusion criteria of the recruitment process have been described in detail previously (24). Two of the subjects withdrew during the intervention, one from the HIIT group due to exercise-induced hip pain and one from MICT group due to personal reasons. This left 13 subjects in each group. The purpose, nature, and potential risks involved in participating in the study were explained in detail, and informed consent was obtained before any measurements were performed. The study was approved (NCT01344928) by the local ethical committee of the Hospital District of South-Western Finland (decision 95/180/2010 §228) and carried out in compliance with the Declaration of Helsinki.

#### Study design.

**Fig. 1.**
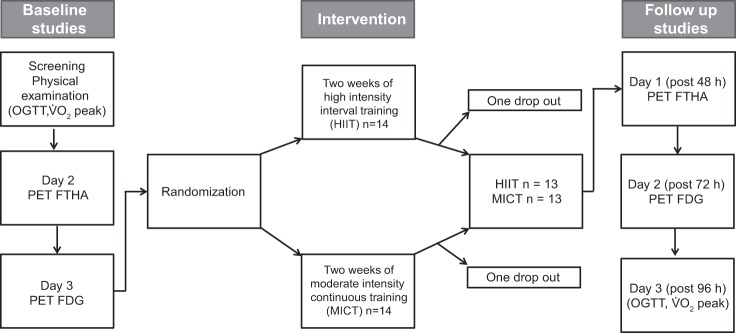
Study design. Subjects were studied on 3 separate days before and after the exercise intervention. OGTT, oral glucose tolerance test; PET, positron emission tomography; FTHA, 14(*R*,*S*)-[^18^F]fluoro-6-thia-heptadecanoic acid ([^18^F]FTHA); PET-FDG, [^18^F]fluoro-2-deoxy-d-glucose ([^18^F]FDG).

Initial screening included a physical examination, an oral glucose tolerance test (OGTT), and a V̇o_2peak_ test to assess the participant’s health, glycemic status, and aerobic capacity. The participants then underwent two PET imaging sessions on 2 different days. On the first day, [^18^F]FTHA and PET were used to measure, under a fasting state, the free fatty acid uptake in different intestinal regions (duodenum, jejunum, and colon) and the quadriceps femoris (QF) and deltoid muscles [the muscle results were taken from our laboratory's previous publication (7)]. On the second day, [^18^F]FDG and PET were used to measure the insulin-stimulated GU in the intestine and the muscles during hyperinsulinemia. Once again the muscle results used were from our laboratory's previous publication (7). An overnight fast of at least 10 h was required before the OGTT and PET measurements. Participants were also asked to abstain from any caffeinated and alcoholic beverages, and to avoid strenuous exercise 48 h before these studies. After the 2-wk exercise training intervention, all measurements were repeated, starting with [^18^F]FTHA PET 48 h after the last exercise session and continuing with a [^18^F]FDG PET after 72 h, and finally an OGTT and V̇o_2peak_ test were done after 96 h (Fig. 1).

#### Exercise interventions.

Participants were randomized into HIIT and MICT exercise groups, and both training groups had six supervised training sessions within 2 wk. Each HIIT session consisted of 4–6 × 30 s exercise bouts of all-out cycling efforts (Wingate protocol, load 7.5% of the whole body weight, Monark Ergomedic 828E, Monark, Vansbro, Sweden) with 4 min of recovery in between the exercise bouts (5). All of the participants were familiarized with the HIIT training protocol (2 × 30 s bouts) before they were randomized into training groups. MICT training consisted of 40–60 min of cycling at a moderate intensity (60% of V̇o_2peak_ intensity). In both groups, the training was progressive, and in the HIIT group the number of cycling bouts increased from four to five and finally to six, and in the MICT group the training time increased from 40 to 50 min and then to 60 min in every second training session.

#### PET scans.

Participants underwent four PET sessions: one [^18^F]FTHA PET and one [^18^F]FDG PET before and after the training intervention. Antecubital veins of both arms were cannulated for the PET studies. One catheter was used to inject the radiotracers [^18^F]FTHA and [^18^F]FDG, whereas the other one was for blood sampling. To arterialize the venous blood samples, the arm was heated using an electronically powered cushion. On the first PET scan session, intestinal free fatty acid uptake was measured using [^18^F]FTHA PET in a fasting state. [^18^F]FTHA radiotracer [155 (SE 0.4) MBq] was injected, and dynamic imaging of the abdominal region (frames 3 × 300 s) was acquired, starting, on average, at 46 min after the tracer injection. This was followed by a femoral region scanning (QF) (frames 3 × 300 s), starting ~65 min after the tracer injection. Finally, the shoulder region (deltoid) (frames 3 × 300 s) was scanned, starting ~90 min after the tracer injection. On the second day, intestinal GU was measured using [^18^F]FDG under euglycemic hyperinsulinemic clamp. On average 87 (SE 1) minutes after the start of the clamp, [^18^F]FDG [156 (SE 0.5) MBq] was injected, and similar time frames were acquired, as described earlier for [^18^F]FTHA scans, starting at 49, 70, and 90 min after the tracer injection. Arterialized blood samples were obtained at regular intervals during both the [^18^F]FTHA and [^18^F]FDG scans to measure the plasma radioactivity to calculate the tracer input function. An automatic gamma-counter (Wizard 1480, Wallac, Turku, Finland) was used to measure the plasma radioactivity. A GE Discovery TM ST system (General Electric Medical Systems, Milwaukee, WI) was used to acquire the PET/computerized tomography (CT) images. CT images were acquired for anatomical references.

#### Image analysis.

The imaging data obtained from the PET scanner were corrected for dead time, decay, and photon attenuation, and the images were reconstructed using the 3D-OSEM method. Carimas 2.7 (http://turkupetcentre.fi/) was used to manually draw the regional tubular three-dimensional regions of interest (ROIs) on sections of the descending duodenum, the jejunum, and the transverse colon, using CT images as anatomical reference. The tubular ROIs were carefully drawn to outline the intestinal wall, while avoiding the intestinal contents and external metabolically active tissues (17). From these regional (duodenum, jejunum, and colon) the ROI time activity curves were extracted.

The rate constant (*K_i_*) for the uptake of the radiotracer ([^18^F]FTHA, [^18^F]FDG) into the cells was calculated using tissue time activity curves obtained from the duodenum, jejunum, and colon, and a tracer input function using a fractional uptake rate method, as previously described (17). Regional glucose and free fatty acid uptakes were calculated by multiplying region-specific *K_i_* by the corresponding plasma glucose or free fatty acid concentration, respectively. For GU, the products were further divided by a lumped constant of 1.15 (17), and a recovery coefficient of 2.5 (17) was applied for the colonic GU to take into account the partial volume effect (4, 25). For the duodenal and jejunal GU, no recovery coefficient was needed. The ROIs for the deltoid and QF muscles were drawn as explained previously (7).

#### Maximal exercise test.

As previously described (24), the V̇o_2peak_ was determined by performing an incremental bicycle ergometer test (Ergoline 800s, VIASYS Healthcare, USA) with direct respiratory measurements using a ventilation and gas exchange (Jaeger Oxycon Pro, VIASYS Healthcare, Germany) at the Paavo Nurmi Centre (Turku, Finland). Initial exercise intensity was 50 W, and after every 2 min the exercise intensity was increased by 30 W until volitional exhaustion. V̇o_2peak_ was expressed as the highest 1-min mean oxygen consumption. The workload at the last 2 min of the test was averaged and used as a measure for maximal performance. The peak respiratory exchange ratio was ≥1.15 and peak blood lactate concentration, measured from capillary samples obtained immediately and 1 min after exhaustion (YSI 2300 Stat Plus, YSI Life Sciences, USA), was ≥8.0 mmol/l for all the tests. A peak heart rate (RS800CX, Polar Electro, Kempele, Finland) within 10 beats of the age-appropriate reference value (220 – age) was true in all except one participant in both groups and in both pre- and posttraining tests. Therefore, the highest value of oxygen consumption was expressed as V̇o_2peak_ and not maximal O_2_ uptake (V̇o_2max_).

#### The euglycemic hyperinsulinemic clamp.

The euglycemic hyperinsulinemic clamp technique was used as previously described (7). Insulin was infused at a rate of 1 mU·kg^−1^·min^−1^ (Actrapid; Novo Nordisk, Copenhagen, Denmark), and blood samples were taken every 5–10 min to adjust the exogenous glucose infusion and to maintain the plasma glucose concentration as closely as possible to the level of 5 mmol/l. Euglycemic hyperinsulinemic clamp was performed after the subjects had fasted at least for 10 h. Insulin (Actrapid, 100 U/ml, Novo Nordisk, Bagsvaerd, Denmark) infusion was started with the rate of 40 mU·min^−1^·m^−2^ during the first 4 min. After 4 min and up to 7 min, infusion rate was reduced to 20 mU·min^−1^·m^−2^, and, after 7 min to the end of the clamp, it was kept constant at 10 mU·min^−1^·m^−2^. Glucose infusion was started 4 min after the start of the insulin infusion with a rate of subject’s weight (kg)·0.1^−1^·g^−1^·h^−1^. At 10 min, glucose infusion was doubled, and after that it was adjusted further according to plasma glucose levels to maintain the steady-state level of 5 mmol/l. Arterialized venous blood samples were collected before the clamp and every 5–10 min to measure the plasma glucose concentration for adjusting the glucose infusion rate. Arterialized plasma glucose was determined in duplicate by the glucose oxidase method (Analox GM9 Analyzer; Analox Instruments, London, UK). Whole body insulin-stimulated GU rate (M-value) was calculated from the measured glucose values collected when the subjects had reached the steady state during the PET scan that was started 87 min (SE 1) after the start of the clamp. FDG-PET study was performed when the subject had reached the stable glucose concentrations at the level of 5 mmol/l (within 5% range for at least 15 min) after positioning into the PET scanner.

#### MRI.

Adipose tissue depot masses were measured with MRI. MRI scans were performed using Philips Gyroscan Intera 1.5 T CV Nova Dual scanner (Philips Medical Systems). Abdominal area axial T1 weighted dual fast field echo images (echo time 2.3 and 4.7 ms, repetition time 120 ms, slice thickness 10 mm without gap) were obtained. To measure different adipose tissue masses, the images were analyzed using SliceOmatic software version 4.3 (http://www.tomovision.com/products/sliceomatic.html). To obtain the mass, the pixel surface area was multiplied with the slice thickness and the density of adipose tissue, 0.9196 kg/l (1).

#### Other measurements.

A 2-h, 75-g OGTT was conducted after the subjects had fasted for 12 h. Blood samples were collected at 0, 15, 30, 60, 90, and 120 min after the glucose ingestion to determine the glucose and insulin levels. Measurements of oxidized low-density lipoprotein (LDL) and oxidized high-density lipoprotein (HDL) were based on spectrophotometric analyses of oxidized lipids in lipoproteins isolated by precipitation methods (2). Whole body fat percentage was measured at the Paavo Nurmi Centre using a bioimpedance monitor (InBody 720, Mega Electronics, Kuopio, Finland).

#### Animal study design.

Twenty-four male Wistar rats were randomly divided into three groups: HIIT (*n* = 8), MICT (*n* = 8), and control (CON) (*n* = 8). At the central animal laboratory of the University of Turku, the animals (aged between 8 and 12 wk) were housed under standard conditions (temperature 21°C, humidity 55 ± 5%, lights on from 6:00 AM to 6:00 PM) with free access to food and tap water. Before the exercise intervention, rodents’ body weight, body fat mass, and lean tissue mass were measured using EchoMRI-700 (Echo Medical Systems LLC, Houston, TX), OGTT and V̇o_2max_ test were performed, and free-living energy consumption measured. Animals in the HIIT and MICT groups had 10 exercise sessions within 2 wk. Each HIIT exercise session was composed of 8–10 × approximately 30 s swimming bouts with 1-min resting period after each bout. Animals in the HIIT group had extra weights of 30–50 g tied to the waist to force them to make all-out efforts. Animals in the MICT group started with 40-min swimming exercise, and thereafter the exercise duration was increased by 10 min every second session until 80 min was reached in the last two sessions. In the MICT group, the rats did not bear any additional weights. One day after the last training session, OGTT was performed, which followed V̇o_2max_ tests on the second and third day after the last exercise session. Thereafter, the animals were kept in the metabolic cages for 2 days. Animals were killed 5 days after from the last exercise session, and intestinal samples from duodenum were collected for protein expression analyses. All animal procedures were approved by the National Animal Experimental Board (ESAVI/5053/04.10.03/2011) and were performed in accordance with the guidelines of the European Community Council Directives 86/609/EEC.

#### Western blot.

The frozen duodenal tissue pieces were homogenized on ice in a lysis buffer (150 mM NaCl, 1% NP-40, 0,5% sodium-deoxycholate, 0,1% SDS, 50 mM Tris·HCl, pH 8.0), supplemented with a protease inhibitor cocktail with an Ultra-Turrax T25 (Ika-Werke). The protein concentration was then quantified with the Thermo Scientific Pierce BCA protein assay kit (Thermo Fisher Scientific) before the sample denaturation with SDS loading buffer containing β-mercaptoethanol (Sigma-Aldrich) in +95°C for 5 min. Samples were run on a 10% SDS–polyacrylamide gel and, after electrophoresis, transferred onto a nitrocellulose membrane (Santa Cruz Biotechnology). An incubation with 5% (wt/vol) milk diluted in Tris-buffered saline-Tween 20 (0,02 M Tris-buffered saline, 0.1% Tween 20) was used to block the unspecific binding sites before the overnight incubation in +4°C with the following primary antibodies: GLUT2 (no. 07–1402, Millipore), CD36 (no. sc-9154, Santa Cruz Biotechnology), vascular endothelial growth factor 2 (VEGFR2) (no. NB-100-530, Novus Biologicals), and β-actin (no. sc-8432, Santa Cruz Biotechnology). The fluorescent signal from the secondary antibodies IRDye 800CW Donkey anti-Rabbit lgG (H^+^L) and IRDye 800CW Donkey anti-Mouse lgG (H^+^L) (LI-COR Biosciences) was detected by using the LI-COR Odyssey CLx Imager (LI-COR). The intensities were normalized to a reference band in each membrane, and the relative values were used for fold-change calculations.

#### Other measurements in rats.

Body composition was measured using EchoMRI-700 (Echo Medical Systems, Houston, TX). Each animal was scanned before and after the exercise intervention, and body fat mass and lean tissue mass were measured. The aerobic capacity was studied by measuring the V̇o_2max_ with rat single-lane treadmill (Panlab- Harvard Apparatus). Animals were familiarized to the rat single-lane treadmill (Panlab-Harvard Apparatus) for 3 days before the V̇o_2max_ test. The test started after a warm-up period. During the test, the angle of the treadmill was 25°, and the speed was increased by 3 cm/s after every other minute until exhaustion. OGTT was performed after 6-h fast. Glucose (20% wt/vol, 1 ml /100 g) was administered orally, and tail vein glucose was measured at 0, 30, 60, 90, and 120 min with a Precision Xceed Glucose Monitoring Device (Abbott Diabetes Care, Abbot Park, IL). Whole body energy expenditure was measured with a metabolic cage (Oxylet system, Panlab, Harvard Apparatus, Spain) over 48 h. The energy expenditure was calculated according to the measured carbon dioxide (CO_2_) production and oxygen (O_2_) consumption and averaged over 24 h.

#### Statistics.

**Fig. 2.**
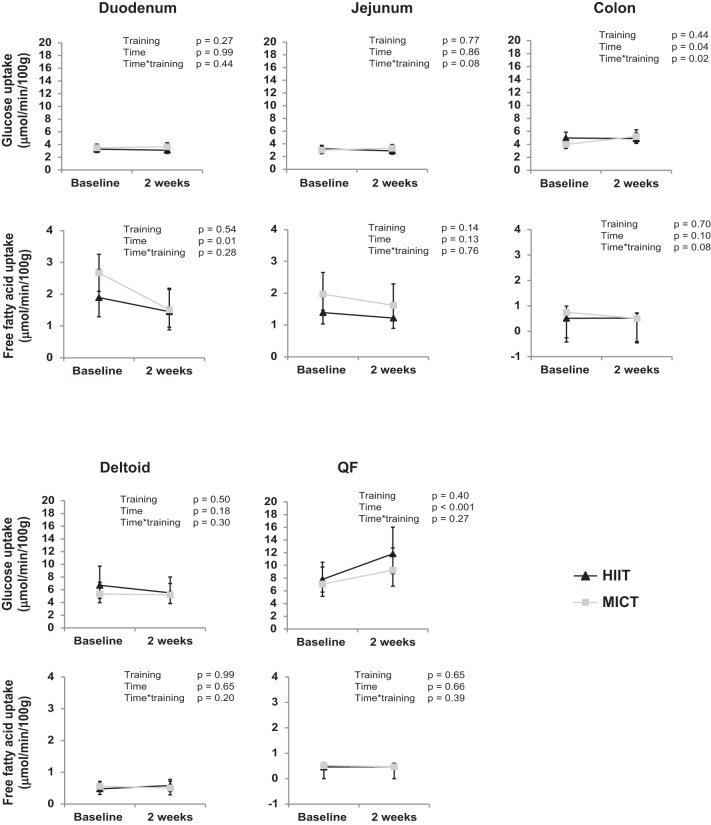
Insulin-stimulated glucose uptake (*top*) and fasting free fatty acid uptake (*bottom*) in different tissues before and after 2 wk of either high-intensity interval training (HIIT; solid triangles) or moderate-intensity continuous training (MICT; shaded squares). The muscle [quadriceps femoris (QF) + deltoid] results have been adapted from Eskelinen et al. (7). All values are expressed as model-based means, and bars are confidence intervals (95% CI). *P* value for time interaction, the groups behaved similarly for the change in parameter with no differences between the training modes. *P* value for time × training interaction, the groups behaved differently for the change in parameter with significant difference between them.

**Fig. 3.**
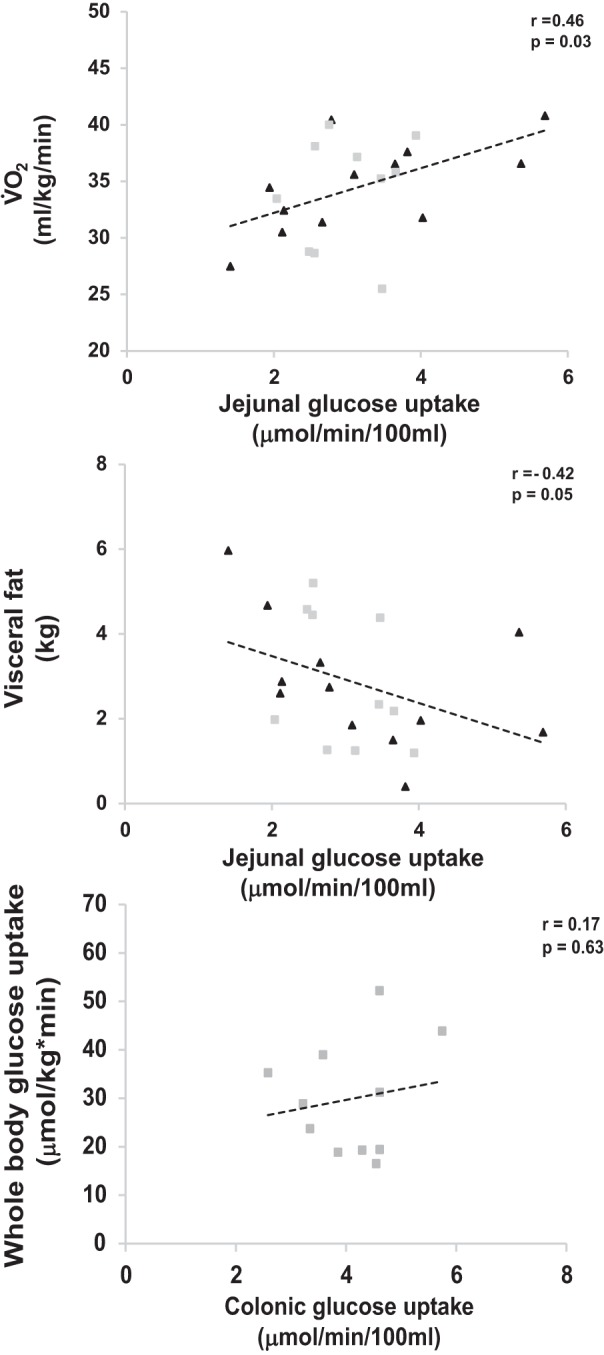
Correlation between insulin-stimulated jejunal glucose uptake and aerobic capacity (V̇o_2peak_; *top*) and visceral fat mass (*middle*) in pooled analysis of moderate-intensity continuous training (MICT; shaded squares) and high-intensity interval training (HIIT; solid triangles) subjects. *Bottom*: correlation between insulin-stimulated colonic glucose uptake and whole body glucose uptake (M-value) in MICT (shaded squares) subjects.

**Fig. 4.**
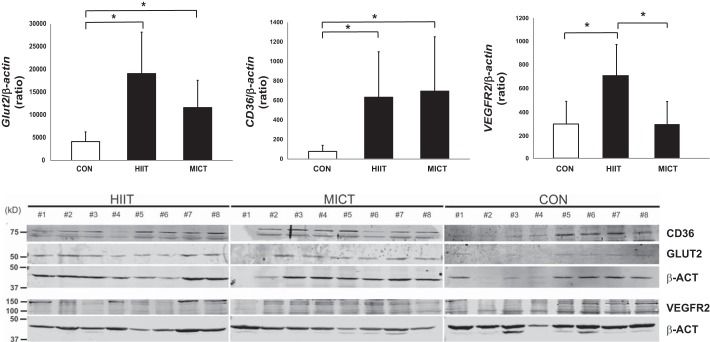
*Top*: relative expression of CD36, GLUT2, and VEGFR2 in duodenum; *n* = 6–8. All values are expressed as model-based means, with error bars representing the confidence intervals (95% CI). *P value < 0.05. *Bottom*: Western blots of CD36 (75 kDa), GLUT2 (55 kDa), and VEGFR2 (105 kDa). Animals without a detectable band were excluded from the analysis. HIIT, high-intensity interval training; MICT, moderate-intensity continuous training; CON, control group.

Descriptive statistics shown in Tables 1 and 2 and Figs. 2–4 are based on model-based means [95% confidence intervals (CI)]. Association between the anthropometrics, glucose profile, and the lipid profile and the training groups, time points, and time × training interaction were performed with hierarchical linear mixed model, using the compound symmetry covariance structure for time. Transformations (logarithmic or square root) were done to (insulin_fasting_; HDL; colonic, QF, and deltoid GU; duodenal, jejunal, colonic, and QF free fatty acid uptake) to achieve the normal distribution assumption. All tests were performed as two-sided, with a significance level set at 0.05. Correlations were calculated using Pearson *r*. In the animal study, one-way analysis of variance was used. All of the analyses were performed using SAS System, version 9.3 for Windows (SAS Institute, Cary, NC).

## RESULTS

### 

#### Characteristics.

The effects of exercise on whole body fat percentage, aerobic capacity (V̇o_2peak_), and whole body insulin sensitivity (M-value) have been published in our laboratory's previous study (7). Total, LDL, and HDL cholesterol levels decreased significantly after training (Table 1). In the cholesterols, the only difference between the training modes was the greater decrease in LDL cholesterol in the HIIT group compared with the MICT group (*P* = 0.03, time × training).

**Table 1.** Subject characteristics at baseline and after the exercise intervention

	HIIT (*n* = 13)			MICT (*n* = 13)			*P* Value	
Parameter	Pre	Post	%Δ	Pre	Post	%Δ	Time	Time × group interaction
Anthropometrics								
BMI, kg/m^2^	25.9 (24.5, 27.3)	25.7 (24.3, 27)	−1	26.4 (25.0, 27.7)	26.4 (25.0, 27.7)	0	0.14	0.19
Whole body fat, %	22.2 (19.8, 24.6)	21.2 (18.8, 23.6)	−5	22.9 (20.5; 25.3)	22.1 (19.7, 24.5)	−3	**<0.0001**	0.56
Subcutaneous fat mass, kg	4.03 (3.3, 4.8)	3.93 (3.2, 4.7)	−2	4.44 (3.7, 5.2)	4.38 (3.6, 5.1)	−1	**0.04**	0.54
Visceral fat mass, kg	2.91 (2.1, 3.8)	2.80 (1.9, 3.7)	−4	2.66 (1.7, 3.5)	2.59 (1.8, 3.4)	−3	**0.046**	0.73
V̇o_2peak_, ml⋅kg^−1^⋅min^−1^	34.7 (32.4, 37.1)	36.7 (34.3, 39.1)	6	33.7 (31.3, 36)	34.7 (32.4, 37.1)	3	**0.001**	0.27
Glucose profile								
Glucose_fasting_, mmol/l	5.5 (5.3, 5.7)	5.4 (5.2, 5.6)	−1	5.7 (5.5, 5.9)	5.6 (5.4, 5.8)	−1	0.43	0.77
Glucose_clamp_, mmol/l	5.0 (4.7, 5.3)	4.9 (4.6, 5.2)	−3	4.9 (4.5, 5.2)	5.0 (4.7, 5.3)	3	0.96	0.20
Insulin_fasting_†, mU/l	5.2 (3.8, 7.2)	4.8 (3.4, 6.6)	−8	5.8 (4.1, 8.1)	6.0 (4.3, 8.5)	4	0.80	0.46
Insulin_clamp_, mU/l	75.3 (66.8, 83.9)	73.8 (65.1, 82.6)	−2	75.4 (66.5, 84.3)	79.4 (70.3, 88.6)	5	0.64	0.31
HbA_1c_, mmol/mol	36.5 (34.3, 38.6)	35.2 (33.0, 37.4)	−4	37.4 (35.3, 39.5)	34.3 (32.1, 36.5)	−8	**<0.001**	0.11
M-value, µmol⋅kg^−1^⋅min^−1^	38.2 (30.1, 46.4)	42.8 (34.5, 51.0)	12	31.9 (23.1, 40.7)	34.2 (25.4, 43.1)	7	**0.03**	0.45
Lipid profile								
FFA_fasting_, mmol/l	0.61 (0.50, 0.71)	0.59 (0.48, 0.70)	−3	0.78 (0.67, 0.89)	0.67 (0.54, 0.79)	−15	0.052	0.14
FFA_clamp_, mmol/l	0.06 (0.05, 0.08)	0.06 (0.05, 0.08)	0	0.08 (0.06, 0.10)	0.07 (0.05, 0.09)	−14	0.41	0.43
Cholesterol, mmol/l	5.3 (4.8, 5.7)	4.6 (4.1, 5.0)	−14	4.7 (4.3, 5.2)	4.4 (3.9, 4.9)	−7	**<0.001**	0.06
HDL†, mmol/l	1.4 (1.2, 1.6)	1.2 (1.1, 1.4)	−10	1.4 (1.2, 1.5)	1.3 (1.1, 1.5)	−5	**<0.001**	0.28
LDL, mmol/l	3.4 (3.0, 3.8)	2.8 (2.4, 3.3)	−16	2.9 (2.5, 2.3)	2.7 (2.3, 3.1)	−6	**<0.001**	0.03
HDL Ox	28.7 (26.3, 31.1)	29.4 (27.0, 31.9)	3	27.4 (24.9, 30.0)	27.6 (25.1, 30.1)	1	0.58	0.74
LDL Ox	30.3 (26.0, 34.5)	31.9 (27.6, 36.1)	5	28.0 (23.6, 32.4)	28.4 (24.0, 32.9)	2	0.26	0.50
Triglycerides, mmol/l	1.02 (0.85, 1.19)	0.97 (0.79, 1.15)	−5	0.96 (0.78, 1.13)	0.80 (0.62, 0.98)	−16	0.07	0.37

Values are means (95% confidence intervals); *n*, no. of subjects. %Δ, percent change. BMI, body mass index; HbA1c, glycosylated hemoglobin; HDL, high-density lipoprotein; LDL, low-density lipoprotein; HDL Ox, oxidized HDL; LDL Ox, oxidized LDL; MICT, moderate-intensity continuous training; HIIT, high-intensity interval training. The *P* value for time indicates the change in the whole study group. The *P* value for time × group interaction indicates if the change in the parameter was different between the HIIT and MICT training modes. Statistically significant values (*P* < 0.05) are bolded.

† Log transformation was done to achieve normal distribution.

#### Intestinal substrate uptake.

Colonic insulin-stimulated GU improved in the MICT group (+37%), while no response was observed in the HIIT group (±0%) (*P* = 0.02 time × training) (Fig. 2). Jejunal GU tended to respond differently between the training modes, with only MICT increasing the uptake (HIIT − 4%, MICT + 13%, *P* = 0.08 time × training) (Fig. 2). Both exercise modes decreased the free fatty acid uptake in the duodenum (*P* = 0.001, time, Fig. 2), and MICT tended to also decrease the uptake in the colon (HIIT 0%, MICT −38%, *P* = 0.08 time × training, Fig. 2). The jejunal GU associated positively with aerobic capacity (V̇o_2peak_) [pretraining (Pre): *r* = 0.46, *P* = 0.03; posttraining (Post): *r* = 0.45, *P* = 0.03] and negatively with visceral fat mass (Pre: *r* = −0.42, *P* = 0.05; Post: *r* = −0.45, *P* = 0.03). GU both in the jejunum (Pre: *r* = −0.31, *P* = 0.15; Post: *r* = −0.50, *P* = 0.02) and duodenum (Pre: *r* = −0.12, *P* = 0.59; Post: *r* = −0.53, *P* = 0.02) associated negatively with HcA1c levels. In the MICT group, the GU in the colon associated positively (Pre: *r* = 0.17, *P* = 0.63; Post: 0.68, *P* = 0.03) (Fig. 3), and the duodenal free fatty acid uptake negatively (Pre: *r* = −0.38, *P* = 0.31; Post: *r* = −0.94, *P* = 0.01), with the whole body GU after the training. QF and deltoid muscle results in these subjects have been published elsewhere (7). For comparison purposes, those results have been added to Fig. 2.

#### Animal results.

There was a significant increase in the body weight and fat free mass of all the animal groups, indicating age-related growth during the study intervention (Table 2). While the fat percentage increased in the CON group, it significantly decreased in both HIIT and MICT groups after the training. There were no differences in glucose values at time points 0 min and 120 min or in the glucose AUC in any of the group × s. The aerobic capacity (V̇o_2max_) tended to improve significantly in both HIIT and MICT groups compared with the CON group [Pre: HIIT: 70.07 (66.2, 74.0); MICT: 71.2 (67.3, 75.1); CON: 69.0 (65.1, 72.9) ml·min^−1^·kg^−0.75^; Post: HIIT: 72.9 (69.0, 76.8); MICT: 72.8 (68.9, 76.7); CON: 68.9 (65.0, 72.8) ml·min^−1^·kg^−0.75^ (95% CI), *P* = 0.05]. GLUT2 protein expression in the rat intestine was significantly higher in the HIIT and MICT groups compared with CON group [HIIT: 19,090 (12,930, 28,190); MICT: 11,606 (7,651, 17,604); CON: 4,141 (2,730, 2,141) arbitrary units (95% CI), *P* < 0.01]. Also, CD36 expression was higher in the HIIT and MICT groups compared with CON group [HIIT: 635 (366, 1,100); MICT: 696 (387, 558); CON: 79 (44, 63) arbitrary units (95% CI), *P* < 0.05]. Whereas VEGFR2 was only higher in the HIIT group compared with MICT and CON groups [HIIT: 704 (477, 976); MICT: 345 (193, 541); CON: 294 (147, 491) arbitrary units (95% CI), *P* < 0.05]. No significant differences were observed in GLUT2, CD36, or VEGFR2 expression between the HIIT and the MICT groups.

**Table 2.** Animal characteristics at baseline and the changes induced after the exercise intervention

	CON (*n* = 8)			HIIT (*n* = 8)			MICT (*n* = 8)			*P* Value	
Parameter	Pre	Post	%Δ	Pre	Post	%Δ	Pre	Post	%Δ	Time	Time × group interaction
Anthropometrics											
Weight, g	282 (269, 294)	351 (338, 364)*	25	297 (285, 309)	346 (331, 360)*	16	281 (269, 293)	350 (337, 364)*	25	**<0.0001**	**0.002**
Fat free mass, %	239 (229, 248)	282 (271, 294)	18	253 (244, 263)	296 (285, 307)	17	248 (238, 257)	291 (279, 302)	17	**<0.0001**	0.99
Fat mass†, g	36.8 (33.6, 40.4)	47.2 (42.2, 52.7)*	28	38.4 (35.0, 42.1)	40.5 (36.3, 45.2)	6	35.9 (32.7, 39.4)	40.4 (36.2, 45.1)*	13	**<0.0001**	**<0.001**
Fat, %	11.9 (11.0, 12.9)	12.7 (11.6, 13.8)*	6	11.7 (10.8, 12.7)	10.7 (9.7, 11.8)*	−8	11.4 (10.4, 12.3)	10.8 (9.7, 11.9)*	−5	0.09	**<.001**
V̇o_2max_, ml·min^−1^·kg^−1^	69.0 (65.1, 72.9)	68.9 (65.0, 72.8)	0	70.1 (66.2, 74.0)	72.9 (69.0, 76.8)*	4	71.2 (67.3, 75.1)	72.8 (68.9, 76.7)	2	**0.01**	**0.05**
OGTT											
Glucose 0, mmol/l	5.0 (4.6, 5.4)	4.9 (4.5, 5.3)	−2	5.1 (4.7, 5.5)	4.9 (4.5, 5.4)	−3	4.9 (4.5, 5.3)	4.7 (4.2, 5.1)	−5	0.31	0.93
Glucose 120, mmol/l	5.5 (5.0, 6.1)	5.3 (4.9, 5.8)	−3	4.8 (4.3, 5.4)	5.2 (4.8, 5.6)	8	5.3 (4.7, 5.8)	4.9 (4.4, 5.3)	−8	0.73	0.23
Glucose AUC, min·mmol^−1^·l^−1^	840 (779, 900)	813 (767, 859)	−3	806 (745, 866)	786 (728, 844)	−2	774 (713, 834)	742 (693, 791)	−4	0.18	0.97

Values are means (95% confidence intervals); *n*, no. of subjects. AUC, area under the curve; CON, control group no exercise; MICT, moderate-intensity continuous training; HIIT, high-intensity interval training. The *P* value for time indicates the change in the whole study group. The *P* value for time × group interaction indicates if the change in the parameter was different between the CON, HIIT, and MICT training modes. Statistically significant values (*P* < 0.05) are bolded.

† Log transformation was done to achieve normal distribution.

* Pre vs. post, *P* value <0.05.

## DISCUSSION

In the present study, the effects of 2 wk of exercise training, HIIT and MICT, on intestinal substrate uptake from circulation were studied in healthy, untrained, middle-aged men. The data show that MICT increases insulin-stimulated GU, while both training modes decrease FFAU in the intestine, and that intestinal insulin-stimulated GU correlates positively with aerobic capacity and negatively with visceral fat and HbA1c. In addition both training modes increased GLUT2 and CD36 protein expressions in rat enterocytes. To our knowledge, this is the first study that provides evidence about the beneficial effects of exercise training on the intestinal substrate metabolism and an additional mechanism by which exercise improves whole body metabolism.

The intestinal GU values during hyperinsulinemia in the present study agree with our laboratory's recent data in healthy lean controls and obese subjects (17, 29). Studies by Honka et al. (17) and Mäkinen et al. (29) show that insulin increases the intestinal GU compared with fasting state in healthy lean controls, but the increase is blunted in obese subjects. This means that the intestine is an insulin-sensitive organ and intestinal insulin resistance exists in obesity. Furthermore, it was shown that, in obese subjects, intestinal insulin resistance is ameliorated after rapid weight loss (17, 29). In enterocytes, glucose is transported from blood to lumen by GLUT2 transporter proteins (40). In obesity and intestinal insulin resistance, there is an impairment in the insulin-stimulated GLUT2 internalization in the enterocyte; which has been suggested to restrain the normal GU in the intestine (41). In the present study, the insulin-stimulated intestinal GU before the training intervention was at the same level as the healthy controls in our laboratory's previous study (29). Insulin-stimulated GU improved in the colon (+37%) and tended to improve in the jejunum in the MICT group after the training, while it remained essentially unchanged in the HIIT group. To study the mechanisms behind the exercise-induced improvements in intestinal GU in our human data, we performed corresponding short HIIT and MICT training interventions in healthy rats. As GLUT2 is responsible for the uptake of glucose from basolateral membrane in the intestine (21), we hypothesized that exercise would increase the expression of GLUT2 in enterocytes to enhance the intestinal GU, and that the increase would be higher in MICT compared with HIIT due to higher training volume. We found that both HIIT and MICT increased intestinal GLUT2 expression in rats with no differences between the groups. The reason why the increased GU was seen only after MICT in humans, whereas GLUT2 expression increased in both training groups in rats, is unclear. However, it might be that, although 2 wk of low-volume HIIT was enough to induce changes in protein level in rats, longer time is need to be able to detect a change in tissue level noninvasively in humans.

The discrepancy in GU in different parts of the intestine agrees with the findings of Mäkinen and coworkers (29) and may be due to the differences in the location of GLUT2 receptor in the enterocytes (41). In humans, GLUT2 has been observed in the apical membrane of an enterocyte in the jejunum, but not in the duodenum (3). The discrepancy in substrate uptake in different parts of the intestine is possibly also related to the different digestive tasks between the small and large intestines and how exercise training strains these mechanisms.

The results in this study demonstrate a decreased free fatty acid uptake in the duodenum after the training intervention in both training groups. The digestion and delivery of dietary fats throughout the body is mediated by the small intestine. In the small intestine, inside the enterocytes, the dietary fats are resynthesized into triacylglycerols (TAG) and secreted into the circulation or stored in cytoplasmic lipid droplets. Postprandially, the increased secretion of TAG from the small intestine leads to an increment in the circulating TAG levels; however, during a fast, the levels decrease as a result of clearance by peripheral tissues (30). Recently, Hung and coworkers (18) showed that, in rodents, endurance training leads to enhanced lipid turnover and more efficient fatty acid oxidation for energy utilization within the enterocytes. Our data regarding the higher CD36 expression, in both HIIT- and MICT-trained rats, is in agreement with the results of Hung et al. (18). Despite the higher CD36 expression, the reduced intestinal FFAU after training in the present study could be due to the more efficient fatty acid oxidation. This is because enhanced fatty acid oxidation means that less fatty acids are needed to produce the same amount of energy.

Another possible mechanism for the decreased intestinal FFAU could be the reduced free fatty acid flux in the intestine. In fact, we found in the present study an almost significant (*P* = 0.052, Table 1) drop in the levels of circulating plasma free fatty acids after the training during the FTHA-PET study (fasting). The lower free fatty acid levels can be explained by decreased visceral fat mass and increased whole body insulin sensitivity posttraining, as both reduce the adipose tissue lipolysis and thereby circulating FFAs (Table 1) (31, 34, 38).

At the moment, little is known about the different mechanisms of how exercise training could strain the intestinal metabolism, yet some data exist about exercise and the splanchnic bed. Splanchnic blood flow reduces during dynamic training and as a function of exercise intensity. However, it has been shown that the reduction in splanchnic blood flow during exercise attenuates as a response to long-term training (32, 33). The smaller reduction in splanchnic blood flow during exercise after regular training seems to be related to the enhanced vasodilation and reduced vasoconstriction of splanchnic and renal vasculature, which further could indicate improved nutrient supply and utilization during exercise in a trained state (33). In the present study, we did not measure intestinal blood flow in humans. In rodents, we found higher VEGFR2 (a marker of angiogenesis) expression level in enterocytes in HIIT compared with MICT and CON groups (Fig. 4). Thus angiogenesis could also be one factor explaining the attenuated reduction in the intestinal blood flow shown after exercise training (33). The difference in VEGFR2 levels between the groups in the present study might be due to higher transient reduction of flow into the splanchnic area during HIIT compared with MICT. HIIT is an extremely intense exercise mode, and, during the intervals, the body concentrates to supply blood mainly to the working muscles, which may induce the hypoxic condition in the splanchnic area and further stimulate intestinal angiogenesis. Other possible factors regulating intestinal metabolism could be peristaltic movements and colon transit time (37, 43).

We used two different training modes in this study. These both included six training sessions within an intervention period of 2 wk. Both the time spent during the training (time HIIT 15 min vs. MICT 300 min) and the average calculated energy consumption during the training [403 and 2680 kcal, respectively (7)] were much less in HIIT than MICT. Despite this difference, both training modes improved whole body insulin sensitivity (M-value, HIIT 12% and MICT 7%) and aerobic capacity (V̇o_2peak_, HIIT 6% and MICT 3%) without significantly different responses between the training modes. In contrast to this, intestinal metabolism seems to be more sensitive to MICT than HIIT. As intestine mediates the delivery of nutrients throughout the body, it may be that the aerobic training mode and longer exercise time per session in MICT compared with HIIT challenges the intestinal metabolism more and thus may be a more rapid and effective way to improve intestinal metabolism.

It is also possible that the difference in the daily habitual physical activity levels or in dietary intake affects the observed findings. In the present study, subjects were instructed not to perform any additional physical activity, except daily normal living, and they reported having done so. However, no pedometer or any other device was used to follow the activity. Thus we cannot completely rule out the possible effect of habitual physical activity on our results. Subjects were also instructed to maintain their normal dietary habits, and they kept dietary logs for 3 days before and during the exercise intervention. According to the dietary logs, there were no changes in the total caloric intake or in the caloric content before and after the intervention in either study group (data not shown).

Most of the beneficial effects of exercise on the whole body are attributed to skeletal muscles, and thus it is interesting to compare these intestinal findings to our laboratory's previous findings concerning skeletal muscles in these same subjects (7). In skeletal muscles, both training modes increased insulin-stimulated GU in the main working muscle, the QF, whereas no changes were observed in deltoid and other upper body muscles (Fig. 2). In addition, no significant changes were observed in the FFAU in any of the studied muscles (7). Adding the findings from the present study to the overall picture, it is interesting to note that intestinal metabolism seems to respond more readily to MICT than the metabolism in the nonworking upper body muscles (Fig. 2).

Previously, intestinal insulin-stimulated GU has been shown to be associated with whole body GU (M-value), in both healthy and obese subjects (17). Our data are in line with these previous findings, showing that whole body GU associates positively with insulin-stimulated GU in the colon and inversely with the duodenal free fatty acid uptake. Furthermore, the jejunal GU correlated positively with the V̇o_2peak_ and negatively with visceral fat mass and HbA1c, which are both known risk markers for metabolic diseases. Thus, although exercise training induces major health benefits through the body’s muscular system, its effects on the intestine, with an average weight of 3–4 kg and surface of 200–300 m^2^, also warrants further research.

There are some limitations in this study. The first is the location of the intestine. This is because, even though the duodenum has a relatively fixed location in the abdomen, the distal segments of the intestine move within the abdomen. This issue was addressed by confirming the drawn ROIs with a CT scan. Second, the results might have been affected by spillover and partial volume effects due to the transaxial resolution of the PET scanner and the thinness of the intestinal mucosal wall. However, this effect was demonstrated to be minimal in our laboratory's previous validation study (17). Third, in this study, we measured the substrate uptake from the circulation into the enterocytes without knowing the release from the enterocytes into the circulation (i.e., from lumen to circulation). Fourth, due to the radiation dose limits, we could not perform the [^18^F]FDG and [^18^F]FTHA PET scans both at fast and during euglycemic hyperinsulinemic clamp. Thus we studied the FFAU at fasting state and GU during euglycemic hyperinsulinemic clamp, in situations when the FFAU and GU, respectively, are at their highest. Finally, the exercise duration in this study was only 2 wk. Although this kind of intervention has been shown to be effective (7, 12, 14, 15, 44), it must be emphasized that the findings show only the early training response and, therefore, the long-term effects of these training modes on intestinal metabolism should be studied further in future experiments.

In conclusion, this study shows that intestinal insulin sensitivity associates positively with aerobic capacity and inversely with the metabolic risk markers visceral adiposity and HbA1C. Two weeks of regular training (HIIT and MICT) were shown to already improve aerobic capacity and whole body insulin sensitivity and, specifically, MICT to induce positive changes in intestinal substrate metabolism in middle-aged, healthy men. The changes in intestinal substrate uptake seem to be related to improvements in GLUT2 and CD36 protein levels. It is likely that regular long-term training has pronounced effects on intestine and whole body metabolism, and thus the role of exercise training on intestinal substrate uptake in patient populations warrant further studies.

## GRANTS

This study was conducted within the Centre of Excellence in Cardiovascular and Metabolic Diseases and supported by the Academy of Finland, the University of Turku, Turku University Hospital, and Åbo Akademi University. The study was financially supported by European Foundation for the Study of Diabetes, the Emil Aaltonen Foundation, the Hospital District of Southwest Finland, the Orion Research Foundation, the Finnish Diabetes Foundation, the Ministry of Education of the State of Finland, the Academy of Finland (Grants 251399, 251572, 256470, 281440, and 283319), the Paavo Nurmi Foundation, the Novo Nordisk Foundation, and the Centre of Excellence funding.

## DISCLOSURES

No conflicts of interest, financial or otherwise, are declared by the author(s).

## AUTHOR CONTRIBUTIONS

K.K.M., J.-J.E., J.T., T.I., M.Y.-K., K.A.V., R.P., E.L., and K.K.K. analyzed data; K.K.M., E.L., K.K.K., and J.C.H. interpreted results of experiments; K.K.M. prepared figures; K.K.M. drafted manuscript; K.K.M., P.N., K.K.K., and J.C.H. edited and revised manuscript; A.M.S., J.-J.E., J.T., T.I., M.Y.-K., K.A.V., J. Kapanen, T.J.G., M.H.-S., O.S., N.S., M.A., K.K.K., and J.C.H. performed experiments; J. Knuuti conceived and designed research; P.N., K.K.K., and J.C.H. approved final version of manuscript.

## References

[1] Abate N, Burns D, Peshock RM, Garg A, Grundy SM Estimation of adipose tissue mass by magnetic resonance imaging: validation against dissection in human cadavers. J Lipid Res 35: 1490–1496, 1994. 7989873

[2] Ahotupa M, Suomela JP, Vuorimaa T, Vasankari T Lipoprotein-specific transport of circulating lipid peroxides. Ann Med 42: 521–529, 2010. doi:10.3109/07853890.2010.510932. 20718696

[3] Ait-Omar A, Monteiro-Sepulveda M, Poitou C, Le Gall M, Cotillard A, Gilet J, Garbin K, Houllier A, Chateau D, Lacombe A, Veyrie N, Hugol D, Tordjman J, Magnan C, Serradas P, Clement K, Leturque A, Brot-Laroche E GLUT2 accumulation in enterocyte apical and intracellular membranes: a study in morbidly obese human subjects and ob/ob and high fat-fed mice. Diabetes 60: 2598–2607, 2011. doi:10.2337/db10-1740. 21852673 PMC3178286

[4] Aston JAD, Cunningham VJ, Asselin MC, Hammers A, Evans AC, Gunn RN Positron emission tomography partial volume correction: estimation and algorithms. J Cereb Blood Flow Metab 22: 1019–1034, 2002. doi:10.1097/00004647-200208000-00014. 12172388

[5] Burgomaster KA, Hughes SC, Heigenhauser GJF, Bradwell SN, Gibala MJ Six sessions of sprint interval training increases muscle oxidative potential and cycle endurance capacity in humans. J Appl Physiol (1985) 98: 1985–1990, 2005. doi:10.1152/japplphysiol.01095.2004. 15705728

[6] Drozdowski L, Thomson AB Intestinal hormones and growth factors: effects on the small intestine. World J Gastroenterol 15: 385–406, 2009. doi:10.3748/wjg.15.385. 19152442 PMC2653359

[7] Eskelinen JJ, Heinonen I, Löyttyniemi E, Saunavaara V, Kirjavainen A, Virtanen KA, Hannukainen JC, Kalliokoski KK Muscle-specific glucose and free fatty acid uptake after sprint interval and moderate-intensity training in healthy middle-aged men. J Appl Physiol (1985) 118: 1172–1180, 2015. doi:10.1152/japplphysiol.01122.2014. 25767035

[8] Everard A, Belzer C, Geurts L, Ouwerkerk JP, Druart C, Bindels LB, Guiot Y, Derrien M, Muccioli GG, Delzenne NM, de Vos WM, Cani PD Cross-talk between *Akkermansia muciniphila* and intestinal epithelium controls diet-induced obesity. Proc Natl Acad Sci USA 110: 9066–9071, 2013. doi:10.1073/pnas.1219451110. 23671105 PMC3670398

[9] Everard A, Cani PD Diabetes, obesity and gut microbiota. Best Pract Res Clin Gastroenterol 27: 73–83, 2013. doi:10.1016/j.bpg.2013.03.007. 23768554

[10] Ferrannini E, Wahren J, Felig P, DeFronzo RA The role of fractional glucose extraction in the regulation of splanchnic glucose metabolism in normal and diabetic man. Metabolism 29: 28–35, 1980. doi:10.1016/0026-0495(80)90094-3. 7351874

[11] Frøsig C, Rose AJ, Treebak JT, Kiens B, Richter EA, Wojtaszewski JFP Effects of endurance exercise training on insulin signaling in human skeletal muscle: interactions at the level of phosphatidylinositol 3-kinase, Akt, and AS160. Diabetes 56: 2093–2102, 2007. doi:10.2337/db06-1698. 17513702

[12] Gibala MJ, Little JP, Macdonald MJ, Hawley JA Physiological adaptations to low-volume, high-intensity interval training in health and disease. J Physiol 590: 1077–1084, 2012. doi:10.1113/jphysiol.2011.224725. 22289907 PMC3381816

[13] Gollnick PD, Saltin B Significance of skeletal muscle oxidative enzyme enhancement with endurance training. Clin Physiol 2: 1–12, 1982. doi:10.1111/j.1475-097X.1982.tb00001.x. 7201906

[14] Guiraud T, Nigam A, Gremeaux V, Meyer P, Juneau M, Bosquet L High-intensity interval training in cardiac rehabilitation. Sports Med 42: 587–605, 2012. doi:10.2165/11631910-000000000-00000. 22694349

[15] Helgerud J, Høydal K, Wang E, Karlsen T, Berg P, Bjerkaas M, Simonsen T, Helgesen C, Hjorth N, Bach R, Hoff J Aerobic high-intensity intervals improve V̇o_2max_ more than moderate training. Med Sci Sports Exerc 39: 665–671, 2007. doi:10.1249/mss.0b013e3180304570. 17414804

[16] Holst JJ, Vilsbøll T, Deacon CF The incretin system and its role in type 2 diabetes mellitus. Mol Cell Endocrinol 297: 127–136, 2009. doi:10.1016/j.mce.2008.08.012. 18786605

[17] Honka H, Mäkinen J, Hannukainen JC, Tarkia M, Oikonen V, Teräs M, Fagerholm V, Ishizu T, Saraste A, Stark C, Vähäsilta T, Salminen P, Kirjavainen A, Soinio M, Gastaldelli A, Knuuti J, Iozzo P, Nuutila P Validation of [^18^F]fluorodeoxyglucose and positron emission tomography (PET) for the measurement of intestinal metabolism in pigs, and evidence of intestinal insulin resistance in patients with morbid obesity. Diabetologia 56: 893–900, 2013. doi:10.1007/s00125-012-2825-5. 23334481

[18] Hung YH, Linden MA, Gordon A, Rector RS, Buhman KK Endurance exercise training programs intestinal lipid metabolism in a rat model of obesity and type 2 diabetes. Physiol Rep 3: e12232, 2015. doi:10.14814/phy2.12232. 25602012 PMC4387752

[19] Irrcher I, Adhihetty PJ, Joseph AM, Ljubicic V, Hood DA Regulation of mitochondrial biogenesis in muscle by endurance exercise. Sports Med 33: 783–793, 2003. doi:10.2165/00007256-200333110-00001. 12959619

[20] Ivy JL Muscle insulin resistance amended with exercise training: role of GLUT4 expression. Med Sci Sports Exerc 36: 1207–1211, 2004. 15235327

[21] Kellett GL, Brot-Laroche E Apical GLUT2: a major pathway of intestinal sugar absorption. Diabetes 54: 3056–3062, 2005. doi:10.2337/diabetes.54.10.3056. 16186415

[22] Kiens B, Essen-Gustavsson B, Christensen NJ, Saltin B Skeletal muscle substrate utilization during submaximal exercise in man: effect of endurance training. J Physiol 469: 459–478, 1993. doi:10.1113/jphysiol.1993.sp019823. 8271208 PMC1143880

[23] Kirwan JP, del Aguila LF, Hernandez JM, Williamson DL, O’Gorman DJ, Lewis R, Krishnan RK Regular exercise enhances insulin activation of IRS-1-associated PI3-kinase in human skeletal muscle. J Appl Physiol (1985) 88: 797–803, 2000. 10658053 10.1152/jappl.2000.88.2.797

[24] Kiviniemi AM, Tulppo MP, Eskelinen JJ, Savolainen AM, Kapanen J, Heinonen IHA, Huikuri HV, Hannukainen JC, Kalliokoski KK Cardiac autonomic function and high-intensity interval training in middle-age men. Med Sci Sports Exerc 46: 1960–1967, 2014. doi:10.1249/MSS.0000000000000307. 24561814

[25] Lammertsma AA, Brooks DJ, Frackowiak RSJ, Beaney RP, Herold S, Heather JD, Palmer AJ, Jones T Measurement of glucose utilisation with [^18^F]2-fluoro-2-deoxy-D-glucose: a comparison of different analytical methods. J Cereb Blood Flow Metab 7: 161–172, 1987. doi:10.1038/jcbfm.1987.39. 3558499

[26] Larsen N, Vogensen FK, van den Berg FWJ, Nielsen DS, Andreasen AS, Pedersen BK, Al-Soud WA, Sørensen SJ, Hansen LH, Jakobsen M Gut microbiota in human adults with type 2 diabetes differs from non-diabetic adults. PLoS One 5: e9085, 2010. doi:10.1371/journal.pone.0009085. 20140211 PMC2816710

[27] Ludvik B, Nolan JJ, Roberts A, Baloga J, Joyce M, Bell JM, Olefsky JM Evidence for decreased splanchnic glucose uptake after oral glucose administration in non-insulin-dependent diabetes mellitus. J Clin Invest 100: 2354–2361, 1997. doi:10.1172/JCI119775. 9410915 PMC508433

[28] Madsen SM, Thorup AC, Overgaard K, Jeppesen PB High intensity interval training improves glycaemic control and pancreatic β cell function of type 2 diabetes patients. PLoS One 10: e0133286, 2015. doi:10.1371/journal.pone.0133286. 26258597 PMC4530878

[29] Mäkinen J, Hannukainen JC, Karmi A, Immonen HM, Soinio M, Nelimarkka L, Savisto N, Helmiö M, Ovaska J, Salminen P, Iozzo P, Nuutila P Obesity-associated intestinal insulin resistance is ameliorated after bariatric surgery. Diabetologia 58: 1055–1062, 2015. doi:10.1007/s00125-015-3501-3. 25631620 PMC4392118

[30] Mansbach CM II, Gorelick F Development and physiological regulation of intestinal lipid absorption. II. Dietary lipid absorption, complex lipid synthesis, and the intracellular packaging and secretion of chylomicrons. Am J Physiol Gastrointest Liver Physiol 293: G645–G650, 2007. doi:10.1152/ajpgi.00299.2007. 17627968

[31] Montague CT, O’Rahilly S The perils of portliness: causes and consequences of visceral adiposity. Diabetes 49: 883–888, 2000. doi:10.2337/diabetes.49.6.883. 10866038

[32] Musch TI, Terrell JA, Hilty MR Effects of high-intensity sprint training on skeletal muscle blood flow in rats. J Appl Physiol (1985) 71: 1387–1395, 1991. 1757362 10.1152/jappl.1991.71.4.1387

[33] Padilla J, Simmons GH, Bender SB, Arce-Esquivel AA, Whyte JJ, Laughlin MH Vascular effects of exercise: endothelial adaptations beyond active muscle beds. Physiology (Bethesda) 26: 132–145, 2011. doi:10.1152/physiol.00052.2010. 21670160 PMC3286126

[34] Polak J, Moro C, Klimcakova E, Hejnova J, Majercik M, Viguerie N, Langin D, Lafontan M, Stich V, Berlan M Dynamic strength training improves insulin sensitivity and functional balance between adrenergic alpha 2A and beta pathways in subcutaneous adipose tissue of obese subjects. Diabetologia 48: 2631–2640, 2005. doi:10.1007/s00125-005-0003-8. 16273345

[35] Richter EA, Nielsen JN, Jørgensen SB, Frøsig C, Birk JB, Wojtaszewski JFP Exercise signalling to glucose transport in skeletal muscle. Proc Nutr Soc 63: 211–216, 2004. doi:10.1079/PNS2004343. 15294032

[36] Simonsen L, Henriksen O, Enevoldsen LH, Bülow J The effect of exercise on regional adipose tissue and splanchnic lipid metabolism in overweight type 2 diabetic subjects. Diabetologia 47: 652–659, 2004. doi:10.1007/s00125-004-1374-y. 15298342

[37] Soffer EE, Summers RW, Gisolfi C Effect of exercise on intestinal motility and transit in trained athletes. Am J Physiol Gastrointest Liver Physiol 260: G698–G702, 1991. 2035639 10.1152/ajpgi.1991.260.5.G698

[38] Stallknecht B, Larsen JJ, Mikines KJ, Simonsen L, Bülow J, Galbo H Effect of training on insulin sensitivity of glucose uptake and lipolysis in human adipose tissue. Am J Physiol Endocrinol Metab 279: E376–E385, 2000. 10913038 10.1152/ajpendo.2000.279.2.E376

[40] Thorens B Glucose transporters in the regulation of intestinal, renal, and liver glucose fluxes. Am J Physiol Gastrointest Liver Physiol 270: G541–G553, 1996. 8928783 10.1152/ajpgi.1996.270.4.G541

[41] Tobin V, Le Gall M, Fioramonti X, Stolarczyk E, Blazquez AG, Klein C, Prigent M, Serradas P, Cuif MH, Magnan C, Leturque A, Brot-Laroche E Insulin internalizes GLUT2 in the enterocytes of healthy but not insulin-resistant mice. Diabetes 57: 555–562, 2008. doi:10.2337/db07-0928. 18057092

[42] Turcotte LP, Swenberger JR, Tucker MZ, Yee AJ Training-induced elevation in FABP(PM) is associated with increased palmitate use in contracting muscle. J Appl Physiol (1985) 87: 285–293, 1999. 10409586 10.1152/jappl.1999.87.1.285

[43] Van Nieuwenhoven MA, Brouns F, Brummer RJ The effect of physical exercise on parameters of gastrointestinal function. Neurogastroenterol Motil 11: 431–439, 1999. doi:10.1046/j.1365-2982.1999.00169.x. 10583850

[44] Wisløff U, Ellingsen Ø, Kemi OJ High-intensity interval training to maximize cardiac benefits of exercise training? Exerc Sport Sci Rev 37: 139–146, 2009. doi:10.1097/JES.0b013e3181aa65fc. 19550205

